# Knowledge, attitudes and practices of patients and healthcare professionals regarding oral health and COPD in São Paulo, Brazil: a qualitative study

**DOI:** 10.1038/s41533-021-00235-x

**Published:** 2021-05-04

**Authors:** Matthew Riley, Amber Swann, Alexander J. Morris, Sonia M. Martins, Rachel Adams, Rachel E. Jordan

**Affiliations:** 1 College of Medical and Dental Sciences, University of Birmingham, Edgbaston, Birmingham, UK; 2 School of Dentistry, University of Birmingham, Edgbaston, Birmingham, UK; 3 Department of Community Health, Faculty of Medicine of ABC, São Bernardo do Campo, São Paulo Brazil; 4 Respiratory Group, Brazilian Society of Family and Community Medicine, São Bernardo do Campo, São Paulo Brazil; 5 Institute for Applied Health Research, University of Birmingham, Edgbaston, Birmingham, UK

**Keywords:** Chronic obstructive pulmonary disease, Lifestyle modification, Inflammatory diseases, Patient education

## Abstract

Poor oral health is associated with worse clinical outcomes in Chronic Obstructive Pulmonary Disease (COPD). This qualitative study aimed to investigate the knowledge, attitudes and practices of COPD patients and primary health care professionals (HCPs) in Brazil - where there are high rates of COPD and periodontal disease. Semi-structured interviews with COPD patients (*n* = 9) and three semi-structured focus groups with HCPs (*n* = 25) were conducted in São Paulo. Interviews were thematically analysed using The Framework Method. Despite a high prevalence of edentulism, patients viewed tooth loss and decay as a norm and neglected preventative oral health practices. HCPs blamed patients for avoiding preventative opportunities, whilst patients discussed significant barriers to oral healthcare. Knowledge of the relationship between oral health and COPD was lacking among HCPs and patients, but all participants were receptive to oral health education. Practitioners identified the need for a COPD primary care pathway that integrates oral health protocols. This study indicates that Brazil must incorporate preventative oral health into COPD management and expand public dental services to increase uptake.

## Introduction

Chronic Obstructive Pulmonary Disease (COPD) is the third leading cause of death among adults in Brazil^1^. Exacerbations are a common and costly complication of COPD, often associated with irreversible loss of lung function, hospitalisation and mortality^2,3^. Aspiration of oral bacteria is a major source of lung microbiota and up to half of COPD exacerbations may result from bacterial infections^4^. Recent evidence suggests an association between COPD and periodontal disease, though the precise nature of any causal relationship is unclear^5–11^. Although the Global Initiative for Chronic Obstructive Lung Disease committee guidelines does not include guidance on managing oral health^11^, evidence suggests that periodontal health differs significantly between frequent and infrequent COPD exacerbators^5,12,13^, with a significant decrease in COPD exacerbation frequency following periodontal treatment^5,7,9^.

These factors are of particular relevance in Brazil. While Brazil’s universal healthcare system was introduced in 1988, oral health was low priority and limited to curative treatment^14,15^. The 2004 Oral Health National Policy aimed to expand access to public services, reduce socioeconomic inequalities, and improve preventative care. However, it was too late for Brazil’s elderly population who had accumulated unmet oral health needs^14^. Over 50% of Brazilian 65-74-year-olds are edentulous and the remainder has some degree of periodontal disease^16,17^.

To date, evidence exploring the attitudes of healthcare professionals (HCPs) towards delivering oral healthcare to COPD patients or the oral health views and practices of COPD patients is sparse. Previous studies suggest that HCPs have limited knowledge of oral-systemic links, but are receptive to interdisciplinary education in primary and secondary care^18–23^. A Chinese study reported that COPD patients have lower oral health knowledge and worse oral health practices than controls^24^. This paper reports qualitative data exploring Brazilian patient and HCP knowledge, attitudes and practices related to oral health and COPD, aiming to provide information to inform the design of future care systems.

## Results

Nine patients took part in the interviews. Two-thirds of the participants were male (67%), with a mean age of 68 years old (Table 1). Five participants were edentulous and four participants reported having at least one natural tooth, though only one had more than 20 teeth—a marker of functional natural dentition^25^. Six participants had not completed Elementary School I (equivalent to <5 years of education).

**Table 1:** Characteristics of interviewed COPD patients.

Characteristic	Mean (years)	Range in years
Age	68	51–78
Time since diagnosed with COPD	2.9	1–7

	*N*	%
Gender		
Female	3	33.3
Male	6	66.7
Race		
White	5	55.6
Black	1	11.1
Red Indian	3	33.3
Education		
Elementary school I incomplete	6	66.7
Elementary school I complete	1	11.1
Elementary school II complete	1	11.1
Bachelor	1	11.1
Employment		
Retired	6	66.7
Neither work or study	2	22.2
Work only	1	11.1
Number of natural teeth		
No natural teeth	5	55.6
1–9 teeth	1	11.1
10–19 teeth	2	22.2
≥ 20 teeth	1	11.1
Prosthesis use		
None	3	33.3
Yes, to replace more than one tooth	1	11.1
Yes, full denture on top	2	22.2
Yes, total dentures (top and bottom)	3	33.3
Grade on MRC dyspnoea scale^a^		
Grade 1	4	44.4
Grade 2	1	11.1
Grade 3	1	11.1
Grade 4	1	11.1
Smoking history		
Ex-smoker	8	88.9
Current smoker	1	11.1

^**a**^Demographic distribution of grade on the MRC scale does not equal 100% as two participants did not disclose their grade.

Twenty-five HCPs were recruited for three focus groups (FG); most participants were female (96%) and between 20 to 40 years old (Table 2). Education level varied, with the most common level being a bachelor’s degree. Four participants reported a special interest or qualification in COPD, although, 12 participants (five nurses, three community health agents (CHA), two oral health assistants (OHA), one dentist and one physiotherapist) reported no regular contact with COPD patients.

**Table 2:** Characteristics of participating family health strategy practitioners.

Characteristic	Focus Group 1 (*n* = 11)		Focus Group 2 (*n* = 7)		Focus Group 3 (*n* = 7)	
	*n*	%	*n*	%	*n*	%
Profession						
General practitioners	2	18.2	—	0	1	14.3
Nurses^**a**^	2	18.2	2	28.6	4	36.3
Community health agents	4	36.3	2	28.6	1	14.3
Dentists	1	9.1	—	0	—	0
Oral health assistants	—	0	1	14.3	1	14.3
Others^**b**^	3	27.2	1	14.3	—	0
Sex						
Male	1	9.1	—	0	—	0
Female	10	90.9	7	100	7	100
Age						
<29	2	18.2	—	0	2	28.6
30–39	3	27.2	4	57	2	28.6
40–49	1	9.1	—	0	—	0
50–59	2	18.2	2	28.6	3	42.9
60–69	1	9.1	—	0	—	0
>70	1	9.1	—	0	—	0
Education level						
Some high school	—	0	1	14.3	—	0
High school	3	27.2	2	28.6	2	28.6
Trade school	1	9.1	1	14.3	3	42.9
Bachelor’s degree	5	45.5	1	14.3	2	28.6
Doctorate or higher	1	9.1	1	14.3	-	0
Duration in role						
1–4 years	1	9.1	3	42	3	42.9
5–9 years	4	36.3	1	14.3	1	14.3
10–19 years	2	18.2	—	0	2	28.6
20+ years	4	36.3	2	28.6	1	14.3
Qualifications or special interest in COPD						
Yes^**c**^	4	36.3	1	14.3	3	42.9
No	7	63.6	5	71.4	4	36.3
Frequency of contact with diagnosed COPD patients						
None	2	18.2	4	57	4	36.3
Every week	3	27.2	—	0	1	14.3
Every month	3	27.2	1	14.3	2	28.6
A few times a year	2	18.2	—	0	—	0
Rarely	1	9.1	1	14.3	—	0

^a^Two nurse participants partly completed the screening forms.

^b^Other professions included: physiotherapist (*n* = 2), nutritionist, speech therapist.

^c^Reported special interests included: Physiotherapist, oncology residence; Doctor, COPD research; others non-specified.

Independent analyses of the two participant groups generated comparable themes and subsequent subthemes (Table 3); all four themes concern both HCPs and patients as the views of each participant group are addressed in every theme.

**Table 3:** Themes and subthemes.

Knowledge, attitudes and practices among COPD patients and healthcare professionals related to oral health and COPD in the primary care setting	
1) Patients perceive dental decay and tooth loss as the norm	
2) Knowledge about the importance of good oral health	A. Knowledge of the association between oral health and COPD B. Inadequate information about good oral health practices
3) Barriers to attending preventative appointments	
4) Proposals for future practice	A. Improved communication of information to patients B. Establish multidisciplinary training and care pathways for oral health

### Theme 1: Patient perceptions of dental decay and tooth loss as the norm

HCPs that had contact with COPD patients reported that their oral health status was often poor. However, all patients viewed their oral health as satisfactory and had no oral health complaints, despite many patients reporting developing tooth decay as young adults, and losing a significant proportion of their teeth.

Both participant groups mentioned that patients did not care about their oral health or tooth loss if it was not painful or did not disrupt daily functions. Therefore, patients completed some daily oral hygiene measures such as tooth brushing, but viewed professional dental care to prevent and manage their dental disease is unnecessary:

*“Sometimes [the dentist] wants to do treatment in a specific tooth, but it is not aching and does not hurt, so I don’t understand why I would do it.” (Patient)*

Instead, patients focused on tooth extractions as the “*fastest or only solution*” (*Patient)* available to relieve symptoms and most viewed dentures as an acceptable replacement to natural teeth:

*“I haven’t got any problems with my teeth because I have a prosthesis now […] Before the dentures, I had lots of toothache and mouth sores so I started extracting my teeth, just going to the dentist and taking them out.” (Patient)*

Patients generally accepted their oral health decline: “*I don’t know why my teeth just got rotten*” (*Patient*). Many viewed oral health as being out of their control and blamed it on external factors, such as tooth loss being “*natural*” (*Patient*) or inherited:

*“[Tooth loss] is a family thing because all of my friends and sisters have all had some of their teeth removed, it’s a common thing.” (Patient)*

Consequently, practitioners felt it was challenging to explain the importance of preventative oral health practices to patients:

*“Usually, patients put all the responsibility and hopes for improving and getting better in healthcare professional. So, when the healthcare professionals give them the responsibility for their health, they just ignore and they don’t care.” (Nurse)*

### Theme 2: Knowledge about the importance of good oral health

Participant knowledge encompasses two important subthemes (Table 3). The following subtheme focuses on knowledge of the association between oral health and COPD.

While all patients showed some awareness of an interconnected relationship between oral and general health, only a minority of patients identified a link between oral and pulmonary health. More specific awareness that dental diseases could be a risk factor for COPD or worsen pre-existing COPD was usually lacking:

*“Interviewer: Do you think your oral health has any impact on your COPD?*

*Patient: The mouth and lungs are two separate things, there is no relation between these*.

*Patient: I think no because my problems are just in the lungs […] there is nothing preventing me from breathing in the mouth.”*

Subsequently, patients had struggled to prioritise oral health and there was an expectation that if a relationship existed, this would have been explicitly explained by healthcare providers:

*“No doctor has ever explained something about oral health and the lungs, so I think there is no connection.” (Patient)*

HCPs were unanimously unaware of any evidence linking COPD and oral health. Nonetheless, practitioners were aware of other oral-systemic relationships due to dental referral protocols at the Basic Health Units (BHUs) such as for kidney disease, pregnancy and diabetes. HCPs were able to use their knowledge that the mouth was a “*doorway*” to systemic problems to theorise vague relationships with COPD:

*“It should be connected because most of the patients with lung problems breathe through their mouths, so they are always exchanging things with their environment through their mouths.” (OHA)*

Regarding the second subtheme - inadequate information about good oral health practices, patients recalled no education or referrals from HCPs about their oral hygiene. With a lack of information provision, most patients relied on family members for oral health education:

*“There were no teachers, doctors or dentists, or anybody that could explain how to brush our teeth. So we just did as the elderly did.” (Patient)*

The patients’ comments were echoed in the FGs. Doctors did not routinely examine oral health, meanwhile, nurses and CHAs only educated patients about general oral self-care or referred them to dental services if they had poor oral health – not because of their COPD. This meant that interventions were not delivered in a preventative capacity:

*“In the nurse appointment when I see poor oral health […] I will try to educate the patient like you have to brush your teeth and floss after meals […] I never thought to educate COPD patients to take care of oral hygiene. [group agrees]” (Nurse)*

Inhaler hygiene education, where patients are instructed to use mouthwash following steroid treatment, was the only reported oral education specific to COPD. However, no patients discussed receiving such education.

### Theme 3: Barriers to patients attending preventative appointments

Few patients reported visiting a dentist in the last year, while some reported their last visit over 25 years ago. Patients mainly opted for “*expensive*” private dental care over public services due to sparse information about public dentists, lack of prosthesis offered and long waiting times – “*sometimes you wait for 1, 2 or 3 years to start the treatment”* (*Patient*). By contrast, HCPs mostly felt that public services were adequate at the BHUs and blamed patients for avoiding opportunities, including not attending preventative appointments:

*“But for the healthcare professional that cleans teeth, the prevention part, people don’t show up […] whereas for the dentists it’s always full.” (OHA)*

Patients stated avoiding the dentist due to past “*bad experiences*” (*Patient*), including pain, problems with extractions and ill-fitting dentures. However, poor access to dental care was also highlighted: “*there was no access to dentists, so we hardly ever went” (Patient)*. Particularly, patients from rural areas reported there used to be just a single dentist, sometimes unqualified, located far away. Thus rural communities used homemade methods for preventative oral health care, including herbal mouthwashes, ashes and mud to clean teeth, and creating fillings using cotton.

Some patients also noted that having COPD further limited their access to dental services:

*“I had to go uphill to go the dental appointment, I couldn’t go because the breathing was too much […] [Lying down at the dentist] is bad for me; it would be uncomfortable to breathe.” (Patient)*

These difficulties were recognised by one HCP who felt that some socially vulnerable patients did care about their oral health and blamed service provision:

*“I see people trying, really willing to do dental care but in our public system, dental care is just given in the commercial hours. So, they can’t just go out of their jobs [during work] just to be here. Outside of the public system is too expensive for them. So, they are willing to do it, but they can’t afford it outside of the public system.” (CHA)*

### Theme 4: Proposals for future practice

Proposals for future practice comprises two valuable subthemes (Table 3). The following subtheme discusses improved communication of information to patients.

The majority of patients were receptive to discussing their oral health with a respiratory clinician. These participants would accept verbal information but did not mention wanting to receive written information. Patients suggested they would be responsive to advice from their doctor:

*“If the doctor says something in the mouth is related to lung problems, I would go to the dentist as fast as I could because I know it is important.” (Patient)*

Practitioners recognised the challenge of educating patients about a complex area. Nurses were highlighted as key practitioners to deliver education as they have a high level of patient understanding. Educational groups were frequently suggested to support patients struggling to understand health advice:

*“The idea was to do an educational group […] Once you have started doing it you can identify the patients who are having more problems with understanding and more difficulties interpreting what they are saying. When they identify these patients, they could work directly with them.” (OHA)*

One HCP highlighted the importance of advertising dental care so more patients attend:

*“We could do an advertisement in front of the BHU to call patients to come for dental care.” (OHA)*

The second subtheme centres around establishing multidisciplinary training and care pathways for oral health. Practitioners in medicine and dentistry were receptive to increasing their role in oral health education with COPD patients if provided with more information about the relationship:

*“Permanent education should be given to all the health professionals about the importance of oral health in COPD […] They should always be reminding patients with COPD that they should always be taking care of their oral hygiene, their teeth and they should go to their annual dental appointment.” (Nurse)*

Practitioners frequently highlighted the need to generate a care pathway for COPD that integrates oral health and supports the multi-professional involvement of primary care HCPs. Design recommendations centred around increasing efficiency in COPD and oral health pathology identification, referral and treatment. Specific suggestions included increasing screening and using a checklist at first contact with patients:

*“There could be a kind of questionnaire or checklist, where you ask the patient for symptoms or any other disease, and directly about COPD. Then some questions about the oral health as well, how long ago your last dentist visit was […] you could direct the patient based on their symptoms and other diseases.” (Nutritionist)*

In one focus group, participants mentioned that “*the government is becoming more aware of COPD” (Speech therapist)* and therefore evidence for the relationship between oral health and COPD should be shared with policymakers to help maintain the momentum of primary care pathway development:

*“There is a meeting organised by municipal health secretary with nurses, dentists, physicians of all areas […] If they explain this in this meeting about the relationship between oral health and COPD, they could just spread information among other professionals in all units.” (Doctor)*

## Discussion

This qualitative study provides important insight into the oral health experiences of COPD patients and HCPs, a previously under-researched area. Our findings indicate that in Brazil there is limited awareness of the relationship between oral health and COPD, and an associated lack of oral health prevention in COPD patients. Key recommendations for future practice include education of HCPs and patients, increasing access to services and incorporation of preventative oral health into the newly developing COPD protocol*.*

With 54% of 65-74-year-olds edentulous in Brazil^17^, it was unsurprising to find that a similar proportion of our study population was edentulous. Comparable to previous research conducted with elderly patients in Brazil^26,27^, all patients in this study were satisfied with their oral health despite a high prevalence of edentulism. Societal norms associated with tooth extractions added to patient acceptability of tooth loss. While pain has been documented as the main reason for dental visits in Brazil^28^, this study showed COPD patients focus on pain relief over preventative treatment. This is consistent with previous reports of poor dental hygiene practices and infrequent dental visits among COPD patients^24,29^.

HCPs believed that public preventative services were adequate at the BHUs and blamed patients for actively avoiding opportunities. However, supporting our findings, the insufficient supply of public services, low income and limited education have been previously identified as barriers for elderly Brazilians seeking dental care^30^. Most HCPs failed to recognise COPD patients’ reliance on private oral healthcare due to poor access to public services.

In concordance with previous evidence^19–21,23^, many HCPs in the current study were knowledgeable about the impact of oral health on general health. Conversely, awareness regarding the relationship between oral health and COPD specifically was limited amongst both HCPs and patients. A survey in India also found that only 24% of dentists understood the connection between oral health and respiratory diseases^31^, while a case-control study in China indicated poor oral health knowledge was common among COPD patients^24^. Furthermore, global studies suggest knowledge about oral health and systemic diseases is low in patients with cardiovascular disease and diabetes^32,33^.

In support of guidelines published in the British Dental Journal^34^, this study emphasises that as part of COPD management, better information should be available to patients about oral health and its importance. Further development and research into suitable patient educational resources are required, so that patients receive relevant risk information, oral hygiene instruction and referrals to dentists when appropriate. Education needs to be communicated appropriately as 70% of Brazil’s population has low oral health literacy, leading to difficulties reading printed educational materials^35,36^. Alongside improved education, encouraging and increasing the accessibility of routine dental visits for COPD patients in Brazil would be beneficial, as these have been linked to tooth retention^37^. For this to be accomplished, Brazil needs to expand the coverage of public dental services and allocate more public resources to fund facilities^38^.

As in other global settings where the desire for collaboration has been demonstrated by HCPs^18–21^, practitioners requested training around COPD and oral health. Similar education programmes have been effective in São Bernardo do Campo, by reducing secondary care referral in the year following COPD education^39^. As outlined by the World Health Organisation, inter-professional teams provide better health-services to their community through better case sharing and HCP skill optimisation^40^. Such inter-professional education should begin with students in training to develop a collaborative practice-ready health workforce^40^, this could be improved in Brazil where there is limited overlap in medical and dental training^41,42^. To achieve this, global studies, including in Brazil, have found that incorporating oral health training programmes into a medical curriculum is effective at increasing medical students’ understanding and awareness of oral health^43,44^.

HCPs expressed the need for a primary care pathway that integrated both COPD and oral health, aligning with the current international trend for inter-professional care^45^. This issue reflects the historical separation between medicine and dentistry, which has been reinforced through service provision, education and legislation. Maintaining beliefs about this separation can lead to ignorance of important oral-systemic links to the detriment of COPD patients^45^. At both ends of the global economic spectrum, the reciprocal impact of these two healthcare disciplines needs to be recognised through the promotion and maintenance of oral health to reduce health inequity^45^.

These findings were gathered from a small group of self-selecting participants. Although 96% of the HCPs included in the study were female, all participants available were recruited and this sample is representative of the wider gender distribution of HCPs in Brazil^46^. Whilst the findings cannot be assumed to be generalisable due to the nature of qualitative research^47^, they do provide important insights into local beliefs about COPD and oral health. By providing rich descriptions of the context and methodology of the research, this paper allows readers to judge transferability^48^.

Throughout the study, attention was given to the validity of the research. While data saturation could not be reached due to the COVID-19 pandemic, validity was enhanced by combining data collection from two participant groups and conducting analytical triangulation by co-coding the transcripts^49^. Moreover, through reflexivity, the researchers acknowledged and accounted for how their background (non-Brazilian) and personal biases may have altered the delivery of interviews and interpretation of data^50^.

Using a lay interpreter may threaten the internal validity of the findings, as a lack of research experience could limit their ability to accurately translate^51^. However, local interpreters are more likely to have awareness of the sociocultural background of the participants^52^, which facilitates rapport and helps accurately communicate cross-culturally^52^. By conducting the patient interviews at BHUs and having FGs which included potential hierarchies, social acceptability bias is also more likely^53^. Due to the wide range of participant responses, the impact is likely to be minimal but this cannot be guaranteed.

In conclusion, findings from this study suggest that despite a high prevalence of oral health problems among patients with COPD, patients and HCPs have inadequate knowledge about the relationship between oral health and COPD. A lack of oral health advice in the respiratory setting, alongside poor oral hygiene practices and difficulties accessing free dental care, has worsened the problem. Insights gained from this study demonstrate there is a clear desire for greater integration between medical and dental services to promote preventative oral health. This could be addressed through the development of educational programmes and integrating oral health protocols into the newly developing primary care pathway for COPD patients. Additionally, Brazil must continue to expand and promote public dental services to ensure equity in accessing oral healthcare.

## Methods

This study is reported against COREQ guidelines (Supplementary Table 1).

### Study design

The aim of the current study was to explore the knowledge, attitudes and practices of HCPs and COPD patients regarding their oral health, and to provide information to design future care. Semi-structured interviews with patients diagnosed with COPD and focus groups (FG) with a mixture of HCPs were conducted in primary care in Brazil between February and March 2020. Patient recruitment and data collection were led by AS, HCP recruitment and data collection were led by MR. All aspects of the qualitative methodology were overseen by an expert in qualitative methods (RA). This study was approved by the University of Birmingham Internal Ethics Review Committee (Ref: IREC2019/Student # 1524229 and Student #1636418) and the ABC School of Medicine Ethics Review Committee (Ref: 28309220.7.0000.0082 and 28309120.5.0000.0082).

### Study setting

The study was conducted in five primary care practices, known as Basic Health Units (BHUs), in the São Bernardo do Campo municipality of São Paulo, Brazil. Aside from high COPD prevalence, São Paulo has a high level of socioeconomic inequalities in health and healthcare use^54^.

Primary care is a community-based system in Brazil, as directed by the Family Health Strategy (FHS)^55^. Each BHU, equivalent to a General Practice, has around four FHS teams^55^, formed of medical and dental HCPs, including general practitioners, nurses, community health agents (CHAs), dentists and oral health assistants (OHAs). The FHS teams provide holistic care to families via health promotion, disease prevention, as well as diagnosis, treatment and rehabilitation^55^.

### Study population and recruitment

Convenience sampling was used to recruit participants to the study due to its user-friendly approach and feasibility^56^. When attending the BHU for a COPD related appointment, patients meeting the eligibility criteria (Table 4) were identified, approached and provided written information about the study. HCPs from the FHS primary care teams who met the eligibility criteria (Table 4) were approached during monthly meetings at the BHUs and given information about the study. Interested participants had the opportunity to ask questions via a translator. All participants who proceeded into the study did so voluntarily, gave informed written consent and were informed that AS and MR were non-Brazilian medical students who had received training in collecting qualitative data. A convenient time was arranged to undertake the interviews and FGs in a private room at the BHU.

**Table 4:** Eligibility criteria for participant recruitment.

**Patients**	
Inclusion criteria	Exclusion criteria
Clinical diagnosis of COPD	Patients unable to speak Portuguese fluently
Receiving treatment at the BHU in São Bernardo do Campo	Inability to provide written or oral informed consent
Legal adults in São Bernardo do Campo	Patients with a career in dentistry/medicine –to investigate lay perceptions
**Healthcare professionals**	
Inclusion criteria	Exclusion criteria
Members of primary FHS teams	Non-specified
Practising in São Bernardo do Campo, São Paulo, Brazil	
Involved with COPD patients^a^	

^a^Due to practical reasons and the availability of staff for taking part in FGs, an ethical amendment was obtained to include additional HCPs who are not usually involved in assessing COPD patients. This included two physiotherapists, a speech therapist, and a nutritionist.

### Data collection

Semi-structured, face-to-face interviews with patients and FGs with HCPs were utilised to gain optimum insight into participants’ perceptions of COPD and oral health^57^. All data collection occurred in private rooms at the BHUs and in Portuguese, with the aid of a local interpreter who had experience with qualitative research. The interpreter performed real-time translation after participants spoke, allowing the interviewers to take an active role in discussion^52^.

FGs and interviews were directed by semi-structured topic guides (Supplementary Tables 2 and 3) which were translated into Portuguese, piloted prior to use and further reviewed after the first session. No significant changes were made to the topic guides and no repeat interviews were conducted. All sessions were audio-recorded with consent. The interview and FG lengths, on average 36 and 67 min, respectively, allowed for a sufficiently detailed discussion of all topics without overwhelming participants. To achieve greater rigour in the research a reflexive approach was utilised^58^; immediately after each interview and FG, field notes were documented.

### Data analysis

An inductive approach to thematic analysis using the Framework Method was undertaken, allowing a systematic yet flexible approach, which is appropriate for large data sets, novice qualitative researchers and multi-disciplinary research teams^59^. It comprises five interconnected stages, as described below^60^. AS and MR transcribed the audio recordings within 24 h and repeatedly read the transcripts to enhance immersion^59^.

Patient transcripts were coded by AS and the HCP transcripts by MR. Each began by developing a draft coding framework derived from codes initially identified in the raw data. One transcript from each participant group was coded by both AS and MR, and the early transcripts and the coding frameworks were circulated within the wider team. Differences of opinion in the analysis were discussed and resolved to limit any personal biases the researchers might have, and a final coding framework agreed for indexing the remaining transcripts. Throughout this indexing stage, the transcripts were read and re-read to ensure the findings were a true reflection of the participant voices. Data were then summarised and charted, and separate themes identified for each participant group. The themes from the two studies were found to be very similar, after detailed comparison and discussion within the team the themes for the two sets of data were then merged.

### Reporting summary

Further information on research design is available in the Nature Research Reporting Summary linked to this article.

## Supplementary information

Supplementary Information

Reporting Summary

## Data Availability

The data used and analysed during this study are available from the corresponding author on reasonable request.
