# Supplementary material for: Knowledge, attitudes and practices of patients and healthcare professionals regarding oral health and COPD in São Paulo, Brazil: a qualitative study
Source: NPJ Prim Care Respir Med. 2021 May 4;31:20. doi: 10.1038/s41533-021-00235-x (PMC8096830; doi:10.1038/s41533-021-00235-x)
Supplement: Supplementary file 1 — Supplementary Information [file 41533_2021_235_MOESM1_ESM.docx]

**Supplementary Table 1: COREQ (COnsolidated criteria for REporting Qualitative research) Checklist**

| No. Item | | Guide questions/description | Reported on Page # |
| --- | --- | --- | --- |
| Domain 1: Research team and reflexivity | | | |
| Personal Characteristics | | | |
| 1. Interviewer/facilitator | | Which author/s conducted the interview or focus group? | The first authors AS and MR conducted all the data collection (methods) |
| 2. Credentials | | What were the researcher’s credentials? E.g. PhD, MD | MR and AS – BSc.  AJM – DDS, FDS.  SMM – M.D.  RA – PhD, RGN.  REJ - PhD |
| 3. Occupation | | What was their occupation at the time of the study? | MR and AS – intercalating medical students  AJM – Senior lecturer in dental public health  SMM -  RA – Research fellow in applied health  REJ – Reader in epidemiology and primary care |
| 4. Gender | | Was the researcher male or female? | MR and AJM are male, AS, SMM, RA and REJ are female |
| 5. Experience and training | | What experience or training did the researcher have? | MR and AS received training on conducting qualitative research during their BSc, and additional bespoke training from Nicola Gage on the Framework Method. All aspects of the qualitative methodology were overseen by a trainer in qualitative methods (RA) (methods) |
| Relationship with participants | | | |
| 6. Relationship established | | Was a relationship established prior to study commencement? | No – first contact with participants was during recruitment at the Basic Health Units (methods) |
| 7. Participant knowledge of the interviewer | | What did the participants know about the researcher? e.g. personal goals, reasons for doing the research | It was explained to all participants that the researchers collecting data were medical students (methods) |
| 8. Interviewer characteristics | | What characteristics were reported about the interviewer/facilitator? e.g. Bias, assumptions, reasons and interests in the research topic | Interviewers were non-Brazilian medical student. (methods) |
| Domain 2: study design | | | |
| Theoretical framework | | | |
| 9. Methodological orientation and Theory | | What methodological orientation was stated to underpin the study? e.g. grounded theory, discourse analysis, ethnography, phenomenology, content analysis | Thematic analysis using the Framework Method (methods) |
| Participant selection | | | |
| 10. Sampling | How were participants selected? e.g. purposive, convenience, consecutive, snowball | | Convenience sampling methods (methods) |
| 11. Method of approach | How were participants approached? e.g. face-to-face, telephone, mail, email | | Participants were recruited face-to-face at the Basic Health Units. (methods) |
| 12. Sample size | How many participants were in the study? | | 9 patients, 25 healthcare professionals (abstract) |
| 13. Non-participation | How many people refused to participate or dropped out? Reasons? | | All participants voluntarily took part in the study, no participants withdrew (methods) |
| Setting | | | |
| 14. Setting of data collection | Where was the data collected? e.g. home, clinic, workplace | | In a private room at the Basic Health Units (methods) |
| 15. Presence of non-participants | Was anyone else present besides the participants and researchers? | | An interpreter was present for all interviews (methods) |
| 16. Description of sample | What are the important characteristics of the sample? e.g. demographic data, date | | Characteristics are displayed in Table 1 and Table 2 |
| Data collection | | | |
| 17. Interview guide | Were questions, prompts, guides provided by the authors? Was it pilot tested? | | The researchers developed the topic guides which were piloted prior to use (Supplementary Table 2 and 3) |
| 18. Repeat interviews | Were repeat inter views carried out? If yes, how many? | | No (methods) |
| 19. Audio/visual recording | Did the research use audio or visual recording to collect the data? | | Interviews were audio recorded (methods) |
| 20. Field notes | Were field notes made during and/or after the interview or focus group? | | Yes (methods) |
| 21. Duration | What was the duration of the inter views or focus group? | | Interview average length was 36 minutes  FG average length was 67 minutes (methods) |
| 22. Data saturation | Was data saturation discussed? | | No. Recruitment for interviews was stopped prematurely due to COVID-19 (discussion) |
| 23. Transcripts returned | Were transcripts returned to participants for comment and/or correction? | | N/A due to the language barrier |
| Domain 3: analysis and findings | | | |
| Data analysis | | | |
| 24. Number of data coders | How many data coders coded the data? | | 2 (methods) |
| 25. Description of the coding tree | Did authors provide a description of the coding tree? | | Not explicitly |
| 26. Derivation of themes | Were themes identified in advance or derived from the data? | | Themes were derived directly from the data (results) |
| 27. Software | What software, if applicable, was used to manage the data? | | No software was used |
| 28. Participant checking | Did participants provide feedback on the findings? | | N/A due to the language barrier |
| Reporting | | | |
| 29. Quotations presented | Were participant quotations presented to illustrate the themes/findings? Was each quotation identified? e.g. participant number | | Yes, quotations were identified by participant category e.g. patient or type of healthcare worker (results) |
| 30. Data and findings consistent | Was there consistency between the data presented and the findings? | | Yes (results) |
| 31. Clarity of major themes | Were major themes clearly presented in the findings? | | Yes (results) |
| 32. Clarity of minor themes | Is there a description of diverse cases or discussion of minor themes? | | Yes. Both themes and subthemes were presented. (results). Table 3 represents the themes and subthemes. When appropriate, divergent cases were reported. |

Developed from: Tong A, Sainsbury P, Craig J. Consolidated criteria for reporting qualitative research (COREQ): a 32-item checklist for interviews and focus groups. International Journal for Quality in Health Care. 2007. Volume 19, Number 6: pp. 349 – 357.

**Supplementary Table 2: Topic Guide for Patient Interviews**

| COPD | - In just a couple of sentences, can you tell me a little bit me about your COPD   - How does it impact your health? |
| --- | --- |
| Oral health | - Can you tell me about your oral health?   - Do you have any oral health problems?   - What do you think causes your oral health problems? - How do you look after your oral health?   - Is oral health something you think much about?   - How important is oral health to you? - What challenges do you face in looking after your oral health?   - What sort of support do you need? |
| Oral health and COPD | - Do you think your oral health affects your general health?   - What relationship do you think there is between oral health and general health? - What do you know about oral health and breathing?   - Do you think oral health has any impact on COPD? |
| Dental services | - Tell me about your experiences of using dental services - Where do you find out information about your oral health?   - Have you ever spoken to your dentist about your COPD? - Are there any difficulties you face looking after your oral health because of your COPD? - Do you think there are any areas of dental care that could be improved for you as a COPD patient? |

| Sub-Topics for Debate  90 minutes | |
| --- | --- |
| COPD and Oral Health | What can you tell me about the relationship between oral health and other diseases?  What can you tell me about the relationship between oral health and COPD?   - Are you aware of any research or policies in this area?   What can you tell me about the oral health of your patients?   - Specifically, COPD patients? - What factors are associated with this? (depends on response)   How do you think your COPD patients think of their oral health?   - Why? |
| Management | What is your current practice with regards to oral health management in COPD patients?   - Is oral health something that you think about whilst managing your COPD patients?   Tell me about your experience of managing oral health in COPD patients  Do you know anything about dentists’ role in oral health management of COPD patients?  Do you think there is a healthcare professional best suited to managing oral health in COPD patients? |
| Changing Practice | Do you think there is a need to change oral health management in COPD patients?   - How could oral health management be changed in COPD patients? - How would this be implemented?   How would you feel if you were required to talk to COPD patients about their oral health?  If change was needed in the management of oral health generally how well do you think this would work?   - Specifically, for COPD patients? |
| Closing Remarks | Is there anything else of importance that hasn’t been discussed regarding oral health and COPD that you think is important to mention?  Of all the topics we discussed today, what is the most important for you? |

**Supplementary Table 3: Topic Guide for HCP interviews**
