# Supplementary material for: Knowledge, attitudes and practices of patients and healthcare professionals regarding oral health and COPD in São Paulo, Brazil: a qualitative study
Source: NPJ Prim Care Respir Med. 2021 May 4;31:20. doi: 10.1038/s41533-021-00235-x (PMC8096830; doi:10.1038/s41533-021-00235-x)
Supplement: Supplementary file 2 — Reporting Summary [file 41533_2021_235_MOESM2_ESM.pdf]

nature research

Corresponding author(s): Amber Swann, University of Birmingham

Last updated by author(s): Mar 12, 2021

Reporting Summary

Nature Research wishes to improve the reproducibility of the work that we publish. This form provides structure for consistency and transparency in reporting. For further information on Nature Research policies, see our Editorial Policies and the Editorial Policy Checklist.

Statistics

For all statistical analyses, confirm that the following items are present in the figure legend, table legend, main text, or Methods section.

n/a	Confirmed
☒	☐ The exact sample size (*n*) for each experimental group/condition, given as a discrete number and unit of measurement
☒	☐ A statement on whether measurements were taken from distinct samples or whether the same sample was measured repeatedly
☒	☐ The statistical test(s) used AND whether they are one- or two-sided<br>*Only common tests should be described solely by name; describe more complex techniques in the Methods section.*
☒	☐ A description of all covariates tested
☒	☐ A description of any assumptions or corrections, such as tests of normality and adjustment for multiple comparisons
☒	☐ A full description of the statistical parameters including central tendency (e.g. means) or other basic estimates (e.g. regression coefficient) AND variation (e.g. standard deviation) or associated estimates of uncertainty (e.g. confidence intervals)
☒	☐ For null hypothesis testing, the test statistic (e.g. *F*, *t*, *r*) with confidence intervals, effect sizes, degrees of freedom and *P* value noted<br>*Give P values as exact values whenever suitable.*
☒	☐ For Bayesian analysis, information on the choice of priors and Markov chain Monte Carlo settings
☒	☐ For hierarchical and complex designs, identification of the appropriate level for tests and full reporting of outcomes
☒	☐ Estimates of effect sizes (e.g. Cohen's *d*, Pearson's *r*), indicating how they were calculated

Our web collection on statistics for biologists contains articles on many of the points above.

Software and code

Policy information about availability of computer code

Data collection	n/a
Data analysis	n/a

For manuscripts utilizing custom algorithms or software that are central to the research but not yet described in published literature, software must be made available to editors and reviewers. We strongly encourage code deposition in a community repository (e.g. GitHub). See the Nature Research guidelines for submitting code & software for further information.

Data

Policy information about availability of data

All manuscripts must include a data availability statement. This statement should provide the following information, where applicable:

- Accession codes, unique identifiers, or web links for publicly available datasets
- A list of figures that have associated raw data
- A description of any restrictions on data availability

The data used and analysed during this study are available from the corresponding author on reasonable request.

Field-specific reporting

Please select the one below that is the best fit for your research. If you are not sure, read the appropriate sections before making your selection.

☐ Life sciences ☒ Behavioural & social sciences ☐ Ecological, evolutionary & environmental sciences

For a reference copy of the document with all sections, see nature.com/documents/nr-reporting-summary-flat.pdf

Behavioural & social sciences study design

All studies must disclose on these points even when the disclosure is negative.

Study description	Qualitative study with semi-structured focus groups and interviews
Research sample	Focus groups: primary healthcare practitioners who are members of the Brazilian "Family Health Strategy" teams. Practising in São Bernardo do Campo, São Paulo, Brazil and involved with COPD patients. 24 participants were female, 1 participant was male, age ranged from 20 to 40 years old. The gender distribution of the participants is similar to distribution of primary healthcare professional roles in Brazil, based on national census and cross-sectional data. This population was chosen as primary healthcare physicians are involved in COPD patient continuity of care as well as oral and medical health promotion.<br>Interviews: patients diagnosed with COPD who are receiving treatment at a primary healthcare centre in São Bernardo do Campo, São Paulo, Brazil. There were 9 participants in total, 6 of which were male. The mean age of participants was 68. These demographics reflect similarly to the results of research profiling the COPD population in Brazil. While the aim of the sample size was 12 participants, based on research on data saturation, recruitment had to stop early due to the COVID-19 pandemic.
Sampling strategy	Convenience sampling was used. The sample size was a result of the availability of participants for the study. Further interviews and focus groups were to be conducted, however, due to the COVID-19 pandemic both researchers had to return to the UK earlier than expected and further data collection was canceled. Therefore, while data saturation cannot be guaranteed, the sample size was deemed sufficient as following interim analyses of the data, it was evident that no new additional issues were emerging from the the focus groups and interviews.
Data collection	Semi-structured, face-to-face interviews with patients were conducted by AS. Focus groups with healthcare professionals were moderated by MR. Both were conducted in private rooms at the primary healthcare centres and in Portuguese, with the aid of a local interpreter who had experience with qualitative research. The interpreter performed real-time translation after participants spoke. Focus groups and interviews were directed by semi-structured topic guides which were translated into Portuguese. All sessions were audio-recorded, using Dictaphones, with consent. Audio files were then transcribed by each researcher. AS was also present for all focus groups to make field notes using a pen and paper.
Timing	Data collection was conducted between February and March 2020.
Data exclusions	No data were excluded.
Non-participation	No participants dropped out or declined participation after consenting to take part in the study
Randomization	As this is a qualitative study, there was no randomization and we did not control covariates.

Reporting for specific materials, systems and methods

We require information from authors about some types of materials, experimental systems and methods used in many studies. Here, indicate whether each material, system or method listed is relevant to your study. If you are not sure if a list item applies to your research, read the appropriate section before selecting a response.

Materials & experimental systems

n/a	Involved in the study	
☒	☐	Antibodies
☒	☐	Eukaryotic cell lines
☒	☐	Palaeontology and archaeology
☒	☐	Animals and other organisms
☐	☒	Human research participants
☒	☐	Clinical data
☒	☐	Dual use research of concern

Methods

n/a	Involved in the study	
☒	☐	ChIP-seq
☒	☐	Flow cytometry
☒	☐	MRI-based neuroimaging

Human research participants

Policy information about studies involving human research participants

Population characteristics	See above
Recruitment	When attending the BHU for a COPD related appointment, patients meeting the eligibility criteria were identified, approached and provided written information about the study. Healthcare professionals from the family care teams who met the eligibility criteria were approached during monthly meetings at the primary care centres and given information about the study. Interested participants had the opportunity to ask questions via a translator. All participants who proceeded into the study did so voluntarily, gave informed written consent and were informed that AS and MR were non-Brazilian medical students who had received training in collecting qualitative data. A convenient time was arranged to undertake the interviews and FGs in a private room at the BHU.<br><br>Due to the nature of qualitative research and the inherent selection bias of using a convenience sample, the findings of this study cannot be assumed to be generalisable. By conducting the patient interviews at BHUs and having FGs which included potential hierarchies, social acceptability bias is also more likely. Due to the wide range of participant responses, the impact is likely to be minimal but this cannot be guaranteed. Through reflexivity, the researchers acknowledged and accounted for how their background and personal biases may have altered the delivery of interviews and interpretation of data.
Ethics oversight	This study was approved by the University of Birmingham Internal Ethics Review Committee (Ref: IREC2019/Student # 1524229 and Student #1636418) and the ABC School of Medicine Ethics Review Committee (Ref: 28309220.7.0000.0082 and 28309120.5.0000.0082).

Note that full information on the approval of the study protocol must also be provided in the manuscript.
